# Screen Time and Body Image in Icelandic Adolescents: Sex-Specific Cross-Sectional and Longitudinal Associations

**DOI:** 10.3390/ijerph19031308

**Published:** 2022-01-24

**Authors:** Soffia M. Hrafnkelsdottir, Robert J. Brychta, Vaka Rognvaldsdottir, Kong Y. Chen, Erlingur Johannsson, Sigridur L. Guðmundsdottir, Sigurbjorn A. Arngrimsson

**Affiliations:** 1 Center of Sport and Health Sciences, University of Iceland, 105 Reykjavik, Iceland; vakar@hi.is (V.R.); erljo@hi.is (E.J.); slg@hi.is (S.L.G.); sarngrim@hi.is (S.A.A.); 2 Diabetes, Endocrinology and Obesity Branch, National Institute of Diabetes and Digestive and Kidney Diseases, Bethesda, MD 20892, USA; brychtar@niddk.nih.gov (R.J.B.); chenkong@niddk.nih.gov (K.Y.C.); 3 Department of Sport and Physical Activity, Western Norway University of Applied Sciences, 5063 Bergen, Norway

**Keywords:** screen time, body image, adolescents, longitudinal association, sex-specific analysis

## Abstract

Studies of adolescent body image and screen use are mostly limited to girls, and longitudinal data are scarce. We examined cross-sectional and longitudinal associations between these variables in mid-adolescent boys and girls. Data was collected when participants were at age 15 and 17, by questionnaire and objective measurements (*n* = 152 had complete data). Sex-specific linear regression was used to explore cross-sectional and longitudinal associations of self-reported screen use (total use, and time spent in gaming, TV/DVD/internet-based watching and internet use for communication) and body image, adjusting for vigorous physical activity, symptoms of depression, and body composition. Screen time was negatively associated with body image at both time points, although more strongly at age 15, and for girls only. Gaming and TV/DVD/internet watching was more strongly associated with body image than internet use for communication. Girls with above median screen time at both ages had 14% lower body image score at age 17 than girls with below median screen time at both time points. Our results suggest that screen use is likely to play a role in the development of body dissatisfaction among adolescent females. Limiting screen time may, therefore, help to mitigate body dissatisfaction in adolescent girls.

## 1. Introduction

Body image can be defined as a person’s perceptions, thoughts, and feelings about his/her body [1]. Body dissatisfaction occurs when feelings towards one’s body are negative and disparate from one’s concept of the ideal body [1]. Negative effects of television use and print media on body image have been reported in numerous studies [2,3]. The impact of newer and increasingly popular media sources, including web-browsing and social media, has been studied progressively in recent years. Findings suggest that internet usage, especially appearance-focused social media use and networking, may have adverse effects on perceptions of one’s physical appearance [3,4,5,6]. This is of special concern for adolescents, as they are experiencing major developmental changes and their internet usage has increased exponentially in recent years, largely through smartphones [7].

Screen time may affect health and wellbeing via psychosocial and psychological effects [8]. Social media, television and other screen-based material provides adolescents with diverse opportunities to compare their physical appearance to that of others, elevating the potential for body dissatisfaction [8]. Girls may be more vulnerable than boys since media extensively promotes an unrealistic thin-beauty ideal for women that is unattainable for most females [8,9,10]. Stronger relationship between screen use and body dissatisfaction in adolescent girls as compared to boys has been reported in the literature [6], although results have been somewhat mixed [5]. Screen time is also typically sedentary and higher levels of sedentary behavior are associated with greater adiposity [11,12,13] and lower fitness [13], both of which may result in body image dissatisfaction [14]. Negative feelings towards one’s body may promote unhealthy weight-control behaviors and eating disorders [2] and have also been linked to depression [15] and lower self-esteem [16,17]. Adolescents may be especially vulnerable due to the major physical and psychological changes they undergo during puberty [18], and their extensive use of social media [19]. Younger adolescents might be more at risk compared to older ones as age has been found to be a significant moderator of the relationship between social media use and body image disturbance [5].

The type of screen-based activity preferred by adolescents has been found to differ across sex [20]. Boys tend to favor computer gaming, whereas girls are more likely to use the internet for communicating via social media. Thus, it is important to determine the effects of specific screen use subtypes on body image in a sex-specific manner [21,22]. Most research on screen time and body image has focused on girls [23,24] and composite screen time, and longitudinal data are scarce.

The aim of this study was to examine the relationship between screen time and body image in Icelandic adolescents, both cross-sectionally at ages 15 and 17, and longitudinally between these time points. We sought to identify differential effects of sex and screen-based activity type on body image. We hypothesized that more screen time across the two-year study period would result in less favorable body image at age 17. 

## 2. Materials and Methods

### 2.1. Sample and Data Collection

Four hundred and eleven tenth-grade students (age 15–16 years, 47% boys and 53% girls) from six compulsory schools in metropolitan Reykjavik, Iceland, were invited in the spring of 2015 to participate in the study; 315 (79%) agreed and 244 had complete data for the variables of interest. Two years later, in the spring of 2017, 168 of the 244 agreed to repeat the measurements. Non-participation in the follow up study was largely due to schedule conflicts, relocation, lack of interest, or because the subjects could not be located. Data are presented for 152 participants (95 girls) with complete data at both time points. Participants provided information regarding their socioeconomic background, health and lifestyle by answering a tablet-based questionnaire (in Icelandic) provided by the research team. Objective measurements of body composition were also performed. Written informed consent was attained from all participants and their guardians, and strict procedures were followed to ensure confidentiality. The study was approved by the Icelandic Data Protection Authority, the National Bioethics Committee (VSNb2015020013/13.07), and the Icelandic Radiation Safety Authority.

### 2.2. Measures

#### 2.2.1. Self-Reported Screen Time

Participants reported average daily weekday and weekend hours spent playing computer games, watching TV/DVD/internet material, using the internet for web-browsing/Facebook/e-mail, and using a computer for “other” activities. Each screen time category had the following response options: 1 = “none”, 2 = “about ½ h”, 3 = “1 up to 2 h”, 4 = “2 up to 3 h”, 5 = “3 up to 4 h”, 6 = “4 to 5 h” and 7 = “more than 5 h”. Weekly averages (h/day) for each screen-based activity were computed using the midpoint for each scoring category (5.5 h/day for category 7) and weighted averages for weekdays and weekends. All screen-based activities were then summed for total daily screen time on all days, weekdays, and weekends. These questions have been used previously to evaluate adolescent screen time in the Icelandic population [25]. 

#### 2.2.2. Self-Reported Vigorous Physical Activity 

Participants were asked: “How often, per week, do you perform physical activity that makes you breathe more rapidly or sweat?”, and given the following response options: 1 = “never”, 2 = “less than once a week”, 3 = “once a week”, 4 = “2–3 times a week”, 5 = “4–5 times a week”, 6 = “almost every day”. This question has previously been used to quantify adolescent vigorous physical activity in the Icelandic population [25,26].

#### 2.2.3. Depression 

A 10-item subscale of the Symptom Checklist 90 (SCL-90) [27] was used to assess how often participants had experienced symptoms of depression during the preceding week. Each item included the following response options: 1 = “almost never”, 2 = “seldom”, 3 = “sometimes”, 4 = “often” and 5 = “almost always”, yielding a total score between 10–50 points. The internal consistency of the scale was satisfactory at both baseline and follow-up (Cronbach’s alphas = 0.94 and 0.92, respectively). This subscale of the SCL-90 has previously been employed in studies on mental well-being among Icelandic adolescents [25,28]. 

#### 2.2.4. Global Self-Esteem 

Global self-esteem was assessed using the Rosenberg Self-Esteem Scale [29]. The scale consists of 10 statements, each rated as positive or negative, with four response options: 0 = “strongly agree”, 1 = “somewhat agree”, 2 = “somewhat disagree” and 3 = “strongly disagree”, yielding a total score between 0–30 points. The internal consistency of the scale was satisfactory at both baseline and follow-up (Cronbach’s alphas = 0.91 and 0.93, respectively). The Rosenberg scale has been widely used for evaluating the self-esteem of young people, and its reliability and validity are well documented [30].

#### 2.2.5. Body Image 

Body image was evaluated by five items from the Body and Self-Image subscale of the Offer Self-Image Questionnaire (OSIQ) [31], which were translated into Icelandic. Participants rated how well the following statements described them: “When I think about how I will look in the future, I am happy”, “I frequently feel ugly and unattractive”, “I am proud of my body”, “I am happy with the way my body has changed in recent years”, and “I feel strong and healthy”. A four-point scale was used for all statements, ranging from 1 = “describes me very well” to 4 = “doesn´t describe me at all”. If necessary, responses were recoded such that higher scores reflected more positive body image, yielding a total score between 5–20. The internal consistency of the scale was satisfactory at both baseline and follow-up (Cronbach’s alphas = 0.83 and 0.82, respectively). This 5-item scale, derived on the basis of item analysis of pilot data in 1992 from groups of 9th and 10th graders in Iceland [32], has been verified for validity and reliability [32] and used in previous studies on Icelandic adolescents [28,33,34,35].

#### 2.2.6. Body Composition 

Body mass index (BMI, kg/m^2^) was calculated from standing height (m) measured to the nearest mm with a stadiometer (Seca model 217, Seca Ltd., Birmingham, UK) and body weight (kg) measured to the nearest 0.1 kg on a calibrated scale (Seca model 813, Seca Ltd., Birmingham, UK) with participants wearing light clothing. Measurements were performed at individual schools at age 15 and at the Icelandic Heart Association at age 17. Body fat percentage was derived from whole-body dual energy X-ray absorptiometry (DXA) scans performed on a GE Lunar bone densitometer (GE Healthcare, Madison, WI, USA) by a certified radiologist at the Icelandic Heart Association in Kopavogur, Iceland.

### 2.3. Statistical Analysis

Descriptive summaries are presented as means and standard deviations for continuous variables and frequencies and percentages for categorical variables, for boys and girls separately. Distributional properties of study variables were examined using distribution analyses (histograms with fitted normal distribution, probability plots, box plots). Model performance of all linear regressions was verified by analyzing the output diagnostics (linearity and independence, normality and homoscedasticity of residuals). Sex differences were evaluated by unpaired *t*-tests for continuous variables and chi-square tests for categorical variables. Measures at ages 15 and 17 were compared using paired *t*-tests. Cross-sectional associations between body image and screen time were assessed using linear regression, with both unadjusted models and models adjusted for body fat percentage, depression score, and vigorous physical activity. Covariates were selected based on prior research [13,14,15,25,35,36] and bivariate correlation analysis. Screen time variables included total screen time, game playing, watching TV/DVD/internet material, and internet use (Facebook/web-browsing/e-mail) on all days, and total screen time on weekdays and weekend days. We tested for an interaction between screen time and vigorous physical activity, with respect to body image. Separate regressions were run for boys and girls at ages 15 and 17. Participants were then categorized by total screen use relative to the median at ages 15 and 17 (5.1 h/day and 6.0 h/day, respectively), yielding the following longitudinal groups: above median screen use at both ages (High–High, HH), above the median at 15 and below at 17 (High–Low, HL), below the median at 15 and above at 17 (Low–High, LH), and below median screen use at both ages (Low–Low, LL). Longitudinal regression analysis assessed the relationship between longitudinal screen use and body image at age 17, with LL as the reference group and baseline values (age 15) for all covariates. Cohen´s d was calculated to estimate the effect size of significant associations (d = mean difference between groups, divided by the pooled standard deviation, see https://www.socscistatistics.com/effectsize/default3.aspx) (accessed on 24 November 2021).

Cross-sectional regression results are presented as standardized betas and adjusted R^2^ coefficients to indicate relative strength of associations and percentage of explained variance in body image, respectively. Longitudinal regression results are presented as unstandardized betas. An α < 0.05 was set as the significance threshold in all analyses. SAS statistical software (v9.4, SAS Institute Inc., Cary, NC, USA; www.sas.com (accessed on 24 November 2021)) was used for all statistical work. 

## 3. Results

### 3.1. Characteristics of Participants

Screen time, physical activity and characteristics of participants with complete data are presented in Table 1 and Table 2 (Table 1: cross-sectional data at both ages along with longitudinal comparison between ages 15 and 17 and Table 2: longitudinal data for screen time).

#### 3.1.1. Screen Time

Median value for total daily screen time (total sample) was 5.1 h at age 15 and 6.0 h at age 17 (Table 1). Average total daily screen time was somewhat higher for boys than girls in 2015 (6.0 vs. 5.4 h, *p* = 0.14), but had become equal for the sexes in 2017 (6.4 h), due to a significant increase between time points for girls. Both sexes had higher screen time on weekends than weekdays at age 15 (1.4 h difference, both *p* < 0.0001), but this difference was smaller at age 17 (1.0 h for boys and 0.8 h for girls (both *p* ≤ 0.001)). Girls spent most of their screen time on the internet in 2015, whereas boys preferred game playing. The pattern of activities changed significantly between 2015 and 2017 among boys, who spent more time on the internet and less time playing computer games in 2017. Girls spent a similar amount of time on the various screen time activities at both time points, except that there was a significant increase in “other” activities between 2015 and 2017. As shown in Table 2, about 37% of boys and 28% of girls remained above the median screen time from age 15 to 17 (HH group), while 30% of boys and 33% of girls were below median screen time at both ages (LL group). About 19% of boys and 18% of girls went from above median screen time at age 15 to below at 17 (HL group), while 14% of boys and 21% of girls showed the opposite trend (LH group).

#### 3.1.2. Body Image

As is shown in Table 1, average body image score was significantly higher for boys than girls, both at age 15 (16.4 vs. 14.3, *p* < 0.0001) and age 17 (16.5 vs. 14.6, *p* < 0.0001). The score did not change significantly between time points, for either sex. The average body image score was significantly lower for girls reporting total screen time above the median value at ages 15 and 17 (HH group) compared to girls with total screen time below the median value at both time points (LL group), 13.4 vs. 15.5, or 14% lower for HH girls. For boys, the body image score did not differ between the HH and LL groups.

#### 3.1.3. Covariates

Data for covariates are presented in Table 1. Both sexes reported participating less frequently in vigorous physical activity at age 17 compared to 15, but the decrease was significant for boys only. However, boys reported more physical activity than girls at both time points (although marginal at age 17). Mean depression score was higher for girls than boys at both time points (both *p* < 0.01), but there was a significant increase in the score for boys between age 15 and 17. Girls had higher body fat percentage than boys at both time points (*p* < 0.0001), and there was a significant increase in this parameter for girls only between age 15 and 17. 

#### 3.1.4. Global Self-Esteem

Boys had higher average self-esteem score at age 17 than girls (*p* = 0.03) and borderline higher score at age 15 (*p* = 0.07), (Table 1). The score did not change significantly between time points, for either sex.

### 3.2. Association of Screen Time with Body Image

Linear regression analyses demonstrate that screen time variables were only significantly associated with body image score for girls, both cross-sectionally at ages 15 and 17, and longitudinally between time points.

#### 3.2.1. Cross-Sectional Association at Age 15

Total daily screen time (all days, weekends and weekdays), time spent in game playing and time watching TV/DVD/internet material were all negatively associated with body image score for girls (all *p* < 0.001, Table 3). The effect size was strong, according to the calculated Cohen’s d for the highest 20% as compared with the lowest 20% in total daily screen time (d = 1.11, all days). Associations remained significant, except for total screen time on weekdays, when the data were adjusted for body fat percentage, depression, and vigorous physical activity (all *p* < 0.05). Using the internet for Facebook/e-mail/web-browsing was borderline significant for girls (*p* = 0.06) in the unadjusted model and non-significant in the adjusted model. We did not find a significant interaction between screen time and vigorous physical activity for either sex. Total screen time, body fat percentage and depression score were all significantly related to body image score in the adjusted model, explaining 40% of the variance in body image score.

#### 3.2.2. Cross-Sectional Association at Age 17

As shown in Table 3, total daily screen time (all days and weekends), game playing, internet use (Facebook/web-browsing/e-mail), and watching TV/DVD/internet material, were all negatively associated with body image score for girls (*p* = 0.0002 to 0.03). The effect size was strong, according to the calculated Cohen’s d for the highest 20% as compared with the lowest 20% in total daily screen time (d = 0.81, all days). Associations became non-significant when the data were adjusted for body fat percentage, depression, and vigorous physical activity. Whereas screen time on weekends had the strongest association with body image score, screen time on weekdays was not associated with this outcome. We did not find a significant interaction between screen time and vigorous physical activity for either sex. Vigorous physical activity and depression score were significantly related to body image score in the adjusted model, explaining 31% of the observed variance in body image score. 

#### 3.2.3. Longitudinal Association, at Age 15 to 17

As shown in Table 4, screen time above the median value at both time points (all days), as compared with screen time below the median both years (group HH vs. group LL), was negatively associated with body image score at age 17 among girls (*p* = 0.005). The effect size was of medium strength, according to the calculated Cohen´s d for the HH group compared with the LL group (d = 0.67, all days). The association remained significant after adjustment for baseline (age 15) body fat percentage, depression score, and vigorous physical activity (*p* = 0.03). Adding baseline body image score to the adjusted model did not meaningfully change the results (*p* = 0.04). Further, adding baseline self-esteem to the model did not change the results (*p* = 0.04). This negative association was stronger for screen time on weekends (*p* = 0.0002 for unadjusted model and *p* = 0.002 for adjusted model) but was not observed for screen time on weekdays. We also found a negative association between screen time on weekends and body image for the LH group compared to the LL group (*p* = 0.03 for both unadjusted and adjusted models). 

## 4. Discussion

As smartphones and social networking become nearly ubiquitous in modern culture, a growing body of research continues to find additional adverse impacts of excessive screen time on well-being. Our results add to this literature, confirming a negative association between screen time and body image for adolescent girls and further demonstrating that girls with consistently higher than median screen time over a two-year period rated their body image lower than girls with below median screen time. We also show that greater time spent in gaming and TV/DVD/internet watching was more strongly associated with lower body image than internet use for Facebook/e-mail/web-browsing, and that the strength of the association between body image and screen time varied by gender, age and type of day (weekdays vs. weekends).

We found a consistent, negative cross-sectional relationship between body image score and total screen time and time spent on various screen activities for girls at ages 15 and 17, with a strong effect size. These results were supported by the longitudinal observation (medium effect size) that girls with above median total screen time at both ages had lower body image scores at age 17 than girls with below median screen time at both ages, even after adjusting for potential confounders. Our results broadly agree with previous cross-sectional studies on the association of media and body image [2,3,4] and with the few longitudinal studies on this subject that have, in most cases, found time spent on social network sites [3], and reading magazines and watching TV [2,37] being predictive of body dissatisfaction. Taken together, these findings suggest that media use may play a causal role in the development of body dissatisfaction.

We observed clear sex differences in this study, with no significant associations between screen time and body image for boys, but consistent negative cross-sectional and longitudinal relationship between these variables for girls. Results of the few prior studies that have analysed this topic in both sexes have been mixed. Studies of total screen use [8], computer use in leisure time [23], TV watching [37] and Facebook use [38] have found an association between screen time and body dissatisfaction in girls but not boys. However, as reviewed by Holland and Tiggemann [3], a few studies on social media use and body image have not observed gender differences. A recent review article reported that screen time, especially social media use, was consistently associated with greater body image concerns among both male and female adolescents, although females appeared more negatively affected [6].

The influence of media on body image may be stronger for girls than boys due to the extensive promotion of an unrealistic thin-beauty ideal for women by the media and objectification of the female body in Western societies [9,10]. Studies have shown that girls tend to be more discontent with their body than boys from an early age, and that adolescent girls typically see themselves as overweight even though they are of normal weight [39,40]. In addition, earlier pubertal maturation with increases in body fat among girls has been linked to body dissatisfaction [41,42]. This may result in greater self-monitoring of appearance among girls, potentially with more screen time spent on appearance related issues, leading to higher levels of body dissatisfaction in females than males [3]. Adolescent girls also tend to have lower self-esteem [30,43] and more depressive symptoms [44] than adolescent boys, which may make them more vulnerable to the negative effects of mass media [8,45]. In addition, the quality of social relationships has been found to be positively associated with screen use among boys [46], which may have beneficial influences on their overall mental well-being [8]. Furthermore, in contrast to girls, early developing boys have been found to be more satisfied with their body than their late maturing male peers, as they experience increased strength and endurance [42]. In our study, girls had lower average body image score and higher depression score than boys at both age 15 and 17, and lower self-esteem score at age 17. 

Screen time, both total time and time spent in subcategories, was more strongly associated with body image score at age 15 than 17. This was despite an increase in screen time between age 15 and 17 for girls, whereas body image score remained similar between these ages. These findings are in line with results reported in a recent large meta-analysis regarding increasing age having a weakening effect on the relationship between social media use and body image disturbance [5]. It can be speculated that greater publicity and awareness of the negative effects of unrealistic body standards presented by the media and increased maturity with age could have contributed to our finding. Results from a recent study on adolescent girls suggest that both parental involvement and school environment may play crucial roles in protecting them from the detrimental effects that social media use may have on their body image [47]. Positive mother–adolescent relationship may be especially beneficial in this respect [48].

Total screen time on weekends was more strongly related to body image than total screen time on weekdays, more so at age 17. A possible explanation may be the higher average screen time observed on weekends, and likely longer continuous periods of screen use on these days. This could be especially relevant at age 17, as parental supervision may have decreased between ages 15 and 17. These results agree with a study on Spanish adolescents, that found computer use in leisure time on weekends, but not weekdays, to be associated with body dissatisfaction among girls [23]. 

Game playing was most strongly associated with body image score at both ages, followed closely by watching TV/DVD/internet material, but internet use (Facebook/e-mail/web-browsing) was only associated with body image at age 17. Prior research has focused on the cross-sectional relationship between TV use and body image, and has found inverse association between these variables, explained by the exposure to thin–ideal images and self-objectification [2,49,50]. According to a recent systematic review, female characters in video games are typically objectified and hypersexualized with disproportionate body parts, which may lead to self-objectification in female players [51], more so than television use [52]. Most prior research has found social media use negatively associated with body image [3,4,5,6,53], with a few exceptions [50,54]. Thus, the lack of association between internet use and body image at age 15 came as a surprise but may be due to the phrasing of our question on internet use, which included Facebook but not other social media sites that later became increasingly popular. In addition, the distinction between internet use and watching internet material may have been unclear and perhaps resulted in the former being misclassified as the latter. However, findings reported in a recent meta-analysis study suggest that internet usage may have less adverse effects on perceptions of one´s physical appearance than traditional media [5].

We adjusted for depression, body composition, and vigorous physical activity in our analyses. It is, however, a matter for debate whether some or all of these variables are confounders or intermediates in a causal pathway between screen time and body image dissatisfaction. Depression may also be the result of body dissatisfaction [3,33]. Regardless, total screen time was a significant explanatory variable for body image in girls at age 15, independent of these covariates. This effect of screen time was not observed, however, at age 17, where depression and vigorous physical activity were the only variables significantly associated with body image in the fully adjusted model. Altogether, these results are consistent with screen time having both direct effects on body image and indirect effects via depression and vigorous physical activity. The longitudinal results were independent of the above listed covariates, as well as of baseline body image and self-esteem.

The results of our study may have important practical implications regarding prevention or amelioration of body dissatisfaction. As previously mentioned, negative feelings towards one’s body may promote unhealthy weight-control behaviors and eating disorders [2] and have also been linked to depression [15] and lower self-esteem [16,17]. Our findings suggest that the mitigation of risk factors for body dissatisfaction is particularly important for adolescent females.

The major strength of this study is the longitudinal design that enabled us to evaluate the effects of screen time on body image across the two-year period from age 15 to 17. The cross-sectional and longitudinal results agree, strengthening the credibility of our findings. Our follow-up time of 2 years is, furthermore, longer than that used in the majority of previous studies in this field. Another strength is that analyses were performed separately by sex, which adds to the limited data on sex-based difference in the relationship between screen time and body image. Still another advantage is the available information on the subtypes of screen use and screen time on weekends as well as on weekdays. Finally, the use of DXA measurements yielded accurate information on body composition in terms of body fat percentage, and we therefore did not have to rely on the cruder measure BMI.

A limitation of the present study is the use of self-report for screen time and vigorous physical activity, which may be subject to recall and reporting biases [55]. Further, our questionnaire included separate questions for screen use in four categories, which were summed for total screen time. While this format can provide more detailed information on screen use, summing the answers may have resulted in an over-estimation of the total screen time, as multi-tasking on different screens, such as watching TV and using a smartphone at the same time, has been reported to be quite prevalent in youth [20]. On the other hand, we did not specifically ask about smart-phone use and our question on social media only included Facebook and lacked other social media platforms, which may have led to under reporting of internet use and total screen time. Capping (quantifying) self-reported screen time of more than 5 h/day (highest category) at 5.5 h/day may also have resulted in an underestimation of total screen time and, thereby, weakened the relationship between screen time and body image. As previously mentioned, misclassification of social media use as watching material via the internet may also be present. Finally, it should be noted that body image construction may differ by gender, potentially complicating evaluation of this variable. However, the body image scale used in our study, a 5-item abbreviated Body and Self-Image subscale of the Offer Self-Image Questionnaire, has been validated for male and female adolescents in Iceland, yielding acceptable reliability and validity [32].

## 5. Conclusions

In summary, we found that screen time was associated with lower body image score among girls, both cross-sectionally at age 15 and 17 and longitudinally between these ages. Our results support the notion that screen use plays a role in the development of body dissatisfaction among adolescent girls. Limiting screen time may help to mitigate body dissatisfaction in this group, potentially reducing the risk of developing serious problems regarding health and well-being. Future studies would benefit from using more precise measures of screen time, such as asking participants to estimate their total screen time, as well as to report on the type of device/platform used. Objective measures for screen use would be ideal and are likely to be implemented in the future.

## Figures and Tables

**Table 1:** Characteristics of the study subjects (57 boys and 95 girls) at age 15 and 17.

	15 y		*p* ^a^	17 y		*p* ^a^
	Boys	Girls		Boys	Girls	
Screen time, h/day, mean (SD)						
Days						
All days	6.0 (2.3)	5.4 (2.4)	0.14	6.4 (2.7)	6.4 (2.7) ^b^	0.91
Weekdays	5.6 (2.2)	5.0 (2.5)	0.17	6.1 (2.8)	6.2 (2.9) ^b^	0.80
Weekends	7.0 (2.7) ^c^	6.4 (3.0) ^c^	0.20	7.1 (3.0) ^c^	7.0 (2.9) ^c^	0.79
Activities						
Games	1.9 (1.2)	0.3 (0.8)	**<0.0001**	1.4 (1.2) ^b^	0.4 (1.0)	**<0.0001**
Internet	1.7 (0.9)	2.6 (1.4)	**<0.0001**	2.3 (1.3) ^b^	2.8 (1.2)	**0.03**
Viewing	1.7 (0.9)	1.8 (1.0)	0.46	1.8 (1.0)	1.9 (1.1)	0.33
Other	0.7 (0.8)	0.7 (0.9)	0.70	0.8 (0.9)	1.3 (1.3) ^b^	**0.01**
Vigorous PA, times/week, mean (SD)	5.1 (1.1)	4.6 (1.4)	**0.02**	4.8 (1.3) ^b^	4.4 (1.4)	0.06
Body fat, %, mean (SD)	17.1 (6.2)	30.1 (6.7)	**<0.0001**	17.6 (6.5)	31.2 (7.3) ^b^	**<0.0001**
BMI, mean (SD)	21.3 (2.8)	22.3 (3.4)	0.05	22.4 (3.1) ^b^	23.0 (4.6) ^b^	0.35
Body image score, mean (SD)	16.4 (2.7)	14.3 (3.1)	**<0.0001**	16.5 (2.4)	14.6 (3.1)	**<0.0001**
Depression score, mean (SD)	14.6 (6.9)	20.4 (10.4)	**<0.0001**	16.3 (7.3) ^b^	20.3 (9.2)	**0.006**
Self esteem score, mean (SD)	22.2 (6.3)	20.1 (6.6)	0.07	23.2 (6.1)	20.7 (7.0)	**0.03**

PA = physical activity. ^a^ *p*-value for test of between sex difference, significant values (*p* < 0.05) are in bold. ^b^ *p* < 0.05 between 15 y and 17 y. ^c^ *p* < 0.0001 between weekdays and weekends.

**Table 2:** Participants’ distribution in categories of daily screen time at 15 y to 17 y.

	Boys		Girls	
	*N*	%	*N*	%
Total screen time—all days				
HH	21	36.8	27	28.4
HL	11	19.3	17	17.9
LH	8	14.1	20	21.1
LL	17	29.8	31	32.6
Total screen time—weekends				
HH	20	35.1	26	27.4
HL	11	19.3	17	17.9
LH	11	19.3	23	24.2
LL	15	26.3	29	30.5
Total screen time—weekdays				
HH	22	38.6	24	25.3
HL	10	17.5	19	20.0
LH	7	12.3	19	20.0
LL	18	31.6	33	34.7

HH: above median screen time at 15 y and 17 y. HL: above median screen time at 15 y but below median screen time at 17 y. LH: below median screen time at 15 y but above median screen time at 17 y. LL: below median screen time at 15 y and 17 y.

**Table 3:** Linear relationships between hours of average daily screen time and body image score.

	15 y (2015)				17 y (2017)			
	Boys		Girls		Boys		Girls	
	β	*p* ^a^	β	*p* ^a^	β	*p* ^a^	β	*p* ^a^
Total screen time ^b^—all days ^c^								
Unadjusted	−0.174	0.20	−0.424	**<0.0001**	0.067	0.62	−0.257	**0.01**
Adjusted ^d^	−0.029	0.81	−0.201	**0.03**	0.088	0.49	−0.032	0.74
Total screen time ^b^—weekends								
Unadjusted	−0.135	0.32	−0.400	**<0.0001**	0.024	0.86	−0.378	**0.0002**
Adjusted ^d^	0.041	0.73	−0.210	**0.02**	0.044	0.73	−0.171	0.07
Total screen time ^b^—weekdays								
Unadjusted	−0.182	0.18	−0.378	**0.0002**	0.080	0.55	−0.186	0.07
Adjusted ^d^	−0.060	0.61	−0.158	0.08	0.099	0.43	0.024	0.80
Game playing ^c^								
Unadjusted	−0.158	0.24	−0.396	**<0.0001**	−0.002	0.99	−0.227	**0.03**
Adjusted ^d^	−0.109	0.37	−0.211	**0.02**	−0.043	0.75	−0.129	0.14
Watching TV/DVD/internet material ^c^								
Unadjusted	−0.112	0.40	−0.338	**0.0008**	0.098	0.47	−0.225	**0.03**
Adjusted ^d^	−0.026	0.83	−0.204	**0.03**	0.064	0.62	0.023	0.81
Internet use (Facebook/email/web-browsing) ^c^								
Unadjusted	0.062	0.65	−0.192	0.06	0.137	0.31	−0.223	**0.03**
Adjusted ^d^	0.070	0.54	−0.016	0.86	0.128	0.33	−0.055	0.55

*N* = 57 for boys and *N* = 95 for girls, at both ages. β = standardized regression coefficient. ^a^ *p*-values for body image vs. screen time variables, significant values (*p* < 0.05) are in bold. ^b^ Total screen time (h/day) = game playing + watching TV/DVD/internet material + internet use (Facebook/e-mail/web-browsing). ^c^ Weighted average for weekends and weekdays. ^d^ Adjusted for body fat percentage, depression score, and vigorous physical activity.

**Table 4:** Categorical total daily screen time from age 15 to 17 versus age 17 body image.

	Boys		Girls	
	β	*p* ^a^	β	*p* ^a^
**All days**				
Unadjusted				
HH	0.168	0.83	−2.141	**0.005**
HL	0.791	0.38	−0.607	0.49
LH	0.882	0.38	−0.948	0.26
LL	Ref.		Ref.	
Adjusted ^b^				
HH	−0.060	0.94	−1.705	**0.03**
HL	0.297	0.75	0.058	0.95
LH	0.309	0.77	−0.893	0.28
LL	Ref.		Ref.	
**Weekdays**				
Unadjusted				
HH	0.086	0.91	−1.269	0.13
HL	0.322	0.74	0.097	0.91
LH	0.722	0.51	−0.640	0.47
LL	Ref.		Ref.	
Adjusted ^b^				
HH	−0.179	0.82	−0.694	0.41
HL	−0.138	0.89	1.156	0.22
LH	0.179	0.88	−0.279	0.76
LL	Ref.		Ref.	
**Weekends**				
Unadjusted				
HH	−0.233	0.78	−2.984	**0.0002**
HL	1.048	0.27	−1.491	0.09
LH	1.048	0.27	−1.747	**0.03**
LL	Ref.		Ref.	
Adjusted ^b^				
HH	−0.599	0.48	−2.568	**0.002**
HL	0.610	0.53	−1.363	0.13
LH	0.452	0.66	−1.797	**0.03**
LL	Ref.		Ref.	

β = unstandardized regression coefficient. HH: above median screen time at 15 y and 17 y; 21 boys (36.8%), 27 girls (28.4%). HL: above median screen time at 15 y but below median screen time at 17 y; 11 boys (19.3%), 17 girls (17.9%). LH: below median screen time at 15 y but above median screen time at 17 y; 8 boys (14.1%), 20 girls (21.1%). LL: below median screen time at 15 y and 17 y; 17 boys (29.8%), 31 girls (32.6%). ^a^ *p*-value for body image vs. screen time category, (with LL as a reference category), significant values (*p* < 0.05) are in bold. ^b^ Adjusted for body fat percentage, depression score and vigorous physical activity.

## Data Availability

Datasets for the current study are available from the corresponding author upon reasonable request.
